# The Impact of Wealth Status on Food Intake Patterns in Filipino School-Aged Children and Adolescents

**DOI:** 10.3390/nu11122910

**Published:** 2019-12-02

**Authors:** Imelda Angeles-Agdeppa, Yvonne M. Lenighan, Emma F. Jacquier, Marvin B. Toledo, Mario V. Capanzana

**Affiliations:** 1 Department of Science and Technology-Food and Nutrition Research Institute, Taguig City 1632, Philippines; marvinbtoledo@gmail.com (M.B.T.); mar_v_c@yahoo.com (M.V.C.); 2 Nestlé Research, Vers-Chez-Les-Blanc, 1000 Lausanne 26, Switzerland; yvonne.lenighan@rd.nestle.com (Y.M.L.); emma.jacquier1@rd.nestle.com (E.F.J.)

**Keywords:** wealth status, nutrition, food patterns

## Abstract

Socio-economic status (SES) has an impact on food consumption in developing countries. However, the impact of SES on dietary patterns in Filipino school-aged children is currently unknown. The aim of this study was to fill this knowledge gap, using data from the 2013 National Nutrition Survey. Dietary intake of 11,691 children (6–18 years) were assessed using a 24-h recall. All food and beverages were assigned to one of 85 food groups. Mean daily intake, percent consuming (prevalence), and contribution to energy intake were determined, and stratified by SES, in 6–9 years old, 10–12 years old, and 13–18 years old. Rice was the most consumed food and the primary source of energy across all three age groups, independent of SES. Children of poor SES presented greater consumption of fish, vegetables, fruit, and table sugar (*p* < 0.05). In comparison, children of rich and middle SES presented greater consumption of milk, chicken, pork, sausages, and soft-drinks (*p* < 0.05). In conclusion, SES impacts the type of foods consumed by Filipino children, with children in the poorest households being most at risk of consuming low-variety diets. This analysis could be used to support public health strategies to improve dietary diversity, and potentially nutrient intake, in Filipino children.

## 1. Introduction

The Philippines is currently experiencing a double burden of malnutrition, wherein the prevalence of underweight and stunting is increasing in parallel with obesity. In 2013, 29.1% of children aged 5–10 years were underweight, 29.9% were stunted, with 9.1% overweight, which increased from 6.6% in 2008 [1]. Adequate food and nutrient intake in childhood are vital for optimal growth and development, yet food insecurity affects approximately 70% of Filipino households [2]. A number of factors, including socio-economic status (SES), play a role in determining the nutritional quality of children’s diets [3].

Recent evidence from the 2013 National Nutrition Survey identified inadequate intake of macro- and micronutrients, including protein, total fat, calcium, iron, vitamin C, vitamin A, folate, riboflavin, thiamin, and phosphorus [4]. The prevalence of these nutrient inadequacies was greater in children and adolescents living in a rural location or of a poor SES [4]. This imbalance in nutrients is potentially due to poor dietary diversity due to limited food consumption variation. Kennedy et al. identified greater micronutrient inadequacies in young Filipino children who were consuming a less diverse diet [5].

A number of countries have documented the impact of poor socio-economic status on nutrient and food group intake in children [3,6]. A study of Bangladesh adolescents found that a poorer SES was associated with lower consumption of eggs, meat, milk, and fruit, which corresponded to lower intake of protein, fat, and riboflavin [6]. A more recent study investigated food intake by SES in Brazilian children, and found that children of poorer SES presented greater consumption of fruit and vegetables, while children of richer SES presented greater intake of milk, cheese, and processed meats, with lower intake of treats, including sweets, crisps, and soft drinks [3]. The contrary is observed in high-income countries, wherein children of a high SES consume greater quantities of fruit and vegetables, as part of a “healthier” diet pattern [7].

However, the impact of socio-economic status on food intake in Filipino children and adolescents is currently unknown. Therefore, the aim of this analysis was to describe the main food intake in a Filipino 6 to18 year old population, by SES, using data from the 2013 National Nutrition Survey.

## 2. Materials and Methods

### 2.1. Study Population

The 2013 NNS is a cross-sectional study conducted nationwide in the Philippines. A multi-staged stratified sampling design was used to represent 17 regions encompassing 80 provinces, covering both urban and rural areas. The first stage was the selection of primary sampling units (PSUs), consisting of one barangay or a combination of contiguous barangays with at least 500 households each. From these PSUs, enumeration areas (EAs) with 150 to 200 households were identified, from which housing units were randomly selected. The third and final stage was the random selection of the households, which was the ultimate sampling unit. About 8592 sample households with a response rate of 87.7% were selected for the survey. An overview of the 2013 NNS, including the sample design, weighting procedure, plan, procedure, and analytic guidelines [8], is available elsewhere. The survey sample included a total of 12,012, comprising children (6–9 years old; *n* = 3594), pre-adolescents (10–12 years old; *n* = 2971), and adolescents (13–18 years old; *n* = 5447). However, 321 outliers were excluded from the analysis throughout data processing and, as a result, a total of 11,691 children were included in the current analysis.

The Ethics Committee of Food Nutrition Research Institute (FNRI) approved the survey protocol. All surveyed households provided informed consent prior to participation.

### 2.2. Data Collection

A single 24-h recall [9] was carried out with both the parents/caregiver and each child during household visits. Qualified dietitians recorded all food and beverages that the child had consumed for the previous 24-h period. Common household measurements such as cups, tablespoons, and bowls were used to estimate the amount of each food item or beverage by size or number of pieces. The information was then converted to grams using a portion to weight list for common foods, compiled through actual weighing of the food samples. The information collected included the name and brand of the food items; however, dietary supplements were not incorporated in the dietary survey.

The updated food composition table (FCT) was completed using new food items (325) found in the food consumption survey (NNS, 2013) with their corresponding nutrient content, as outlined below, as well as inclusion of new nutrients of existing food items in the FCT. With this effort, the new Philippine FCT contains 27 nutrients with a total of 1359 food items from the original FCT with 12 nutrients and 1034 food items. Briefly, food composition data were compiled through the use of food matching, recipe calculation, and borrowing of data from other food composition tables (FCTs)/food composition databases (FCDBs) developed by the United States Department of Agriculture (USDA) [10], Association of Southeast Asian Nations (ASEAN) [11], Singapore [12], Thailand [13], Bangladesh [14], Japan [15], Philippine FCT 1997 [16], and the expanded Philippine FCT which follows the FAO/INFOODS Guidelines for Food Matching Version 1.2 [17].

Wealth index was computed based on principal component analysis (PCA) of household assets, household characteristics, access to utilities, and infrastructure variables. The variables collected for the household information were the following: type of dwelling unit, tenure status of the house, tenure status of the lot, type of roof, type of wall, type of floor, number of bedrooms, type of fuel used, transport utilities used (bicycle, motorcycle, car/jeep/van, tractor, caritela, boat), presence of electricity in the household, functioning appliances (computer/laptop/tablet, telephone, cellphone/smartphone, television, radio/cassette recorder, VCD/DVD player, camera/video camera, refrigerator/freezer, stove/range/microwave oven, blender/food processor, electric generator, air-conditioner, washing machine, electric fan, sewing machine, piano/organ, and wall clock) [8]. Weights or factor scores were generated for each household asset/information through PCA. The standardized scores were then used to create the break points that define the wealth quintiles: poorest, poor, middle, rich, and richest.

To simplify the results, in the current analysis, we combined the poorest and poor groups as poor, and the rich and richest groups as rich.

Weight was measured using mechanical Detecto^®^ platform beam balance scales (Detecto, Webb City, Missouri, USA). At least two measurements were obtained, with the average recorded to the nearest 0.1 kg. A third measurement was only taken if the difference between the first two measurements was greater than 0.3 kg. Standing height was obtained for subjects of two years old and over using the Microtoise (SECA 206, Hamburg, Germany)—an L-shaped device (head-bar) to which a spring-loaded coiled tape measure was attached. At least two measurements were obtained, and averages were computed and recorded to the nearest 0.1 cm. A third measurement was only taken if the difference between the first two measurements was greater than 0.5 cm. The waist circumference was measured as the perimeter or distance around the natural waist (midway between the lowest rib and the tip of the hip bone) or a measure of the distance around the narrowest portion of the trunk. The tape measure was placed at the midpoint and the respondent was asked to breathe normally; measurement was taken at the end of normal expiration. Waist and hip circumference was measured among subjects 10 years old and above, excluding pregnant women. The World Health Organization Child Growth Standards (WHO-CGS) was used to assess the nutritional status of children 0 to 5.0 years old (0–60 months), based on weight and height measurements. For the nutritional status of children 5.08 to 19.0 years old (61–228 months), the WHO Growth Reference 2007 was used. The in-depth methods of anthropometric measurements and nutritional status were presented in the 8th NNS Overview [8].

### 2.3. Data Processing

Food coding and amount consumed were reviewed to avoid misclassification and under- or overestimated nutrients intake. For the evaluation of energy intake, the ratio of daily energy intake to the estimated energy requirement (EER) was calculated for each person per day, then transformed to the logarithmic scale to remove outliers below −3 standard deviations (SD) and above +3 SD for each age group [18].

The 17 food components of the Philippine Food Composition Table (PFCT) were reorganized using FITS template and followed the FAO/INFOODS, Analytic Food Composition Database Version 1.1. (FAO, Rome, 2016) [19]. Further, the classifications were designed to be similar to the United States Department of Agriculture (USDA) Food Groupings, but at the same time, to reflect the characteristics of the Filipino diet by traditional way of consumption and frequently consumed foods. To make the results of this study comparable with the information obtained in other countries, and to give a clear picture of the current sources of nutrients, 80 food groups were concluded to reflect the Filipino diet (Table 1).

### 2.4. Statistical Analysis

Stata (Stata Statistical Software, release 15, Stata Copr. 2017) was used to calculate the percentage contribution of each food group to the total energy intake among 6–9, 10–12, and 13–18 years old by socio-economic status. All food and beverages were categorized as one of the 80 food groups. Percent consuming was expressed by percentage of individuals who consumed specific foods or food groups at least once in the 24-h dietary recall, regardless of the amount consumed [20]. The percentage contribution of each food group was calculated by summing the amount of energy by each food group and then dividing by the total energy intake consumed in the total population. The mean food group intake was the average consumption of each food group intake of consumers only. In order to understand food consumption in detail, and change with age and socio-economic status, data for food consumption and food sources of energy are presented for three age groups: 6–9-year-old children, 10–12-year-old pre-adolescents, and 13–18-year-old adolescents by socio-economic classification (poor, middle, and rich).

Due to the assumption of normality, a log-transformation was carried out to deal with skewed data. A one-way ANOVA with Bonferroni multiple comparison adjustment was used to test the difference of mean intake (based on 24-h food recall) of each food group by socio-economic status. An independent *t*-test for two proportions with Bonferroni adjustment was used to test the difference of proportion consuming each food group between wealth statuses.

## 3. Results

### 3.1. Population Characteristics

The characteristics of the child and adolescent study population are presented in Table 2. The overall gender distribution of the sample was approximately 50% boys and 50% girls. Fifty-two percent of the 6–9 years old group were from poor households, of which 66% lived in a rural location, compared to 18% and 16% in the middle and rich SES, respectively. A similar trend was observed in 10–12-year-old children, wherein 55% were of poor SES, of which 68% lived in a rural location. In 13–18-year-old children, 48% were of poor SES, and 32% of rich SES, with 60% of the poor SES dwelling in a rural location, compared to 50% of the rich SES dwelling in an urban location. The percentage of households of middle SES was similar in both rural and urban locations (20%).

Among 6–9-year-old children, the prevalence of being underweight was higher in poor households (40%) as compared to middle (29%) and rich (15%) households. The prevalence of stunting was 39% in poor households as compared to 25% and 13% in middle and rich households. Poor (11%) and middle (11.1%) households were almost equal in terms of the prevalence of wasting, while only 7% in rich households. More overweight children were observed among rich households (9%) compared to poor (2%) and middle (4%) households. Among 10–12 years old and 13–18-year-old adolescents, it is also noticeable that poor households had a higher prevalence of stunting (poor, 39–41%; middle, 26–27%; and rich, 16–19%). Wasting was high in poor (12–14%) and middle (14–16%) households in comparison to rich households (10–14%). A higher proportion of overweight school children and adolescents was seen in rich households (8–12%) than in poor (3%) and middle (4–5%) households.

### 3.2. Food Group Intake: Percent Consuming

#### 3.2.1. 6–9 Year Olds

There were significant differences in the percent of children consuming foods, according to SES categories (Table 3). Rice (any) was consumed by 89% of 6–9-year-old children of poor SES, and 95% of middle SES, which was significantly higher than in the children from the rich households (50%) (*p* < 0.05). Consumption of any fish (66%), any vegetables (53%), and any fruit (19%) was greater in children of poor SES, than in those of middle and rich SES. Any sugar consumption was greater in those of poor SES (29%) than of rich SES (12%) (*p* > 0.05), and also between those of middle and rich SES (*p* < 0.05). Milk (any) consumption was 18% in children of middle and rich SES, compared to 9% in those of poor SES (*p* < 0.05). Consumption of any pork (poor, 14%; middle, 24%; rich, 19%), any chicken (poor, 11%; middle, 20%; rich, 16%), and any sausages (poor, 7%; middle. 19%; rich. 15%) was greater in children of middle and rich SES (*p* < 0.05). Fruit consumption was significantly higher in those of poor (19%) and middle (15%) SES than in those of rich SES (7%). Soft drinks (any) consumption was significantly higher in those of rich (8%) and middle (7%) SES than in those of poor SES (4%).

#### 3.2.2. 10–12 Years Old

An alternate trend in rice consumption was observed in the 10–12-year-old children, wherein 87% of children of poor SES consumed any rice, compared to 99% of children of rich and 94% of middle SES (*p* < 0.05). Fat (any) consumption was significantly higher in the rich (78%) and middle (69%) households than in those of poor (59%) SES. Similar to the 6–9-year-old children, consumption of any fish and any vegetables was greater in children of poor SES (69%) as compared with those of middle (60%) and rich SES (57%) (*p* < 0.05), while any fruit consumption was similar between those of poor (19%) and of rich SES (16%)—but significantly higher in those of poor than of middle SES (13%). In this age group, any egg consumption was greater in children of rich (33%) and middle SES (30%) than in the poor households (20%), while consumption of any bread, any milk, any pork, any sausages, and any chicken was greater in those of rich and middle SES. Table sugar consumption in the poor (27%) was similar with the middle (26%) households, but greater in those of poor SES than of rich SES (22%); yet, consumption of any sugar-sweetened beverages was greater in children of rich (20%) and middle (20%) SES than of poor SES (6%).

#### 3.2.3. 13–18 Years Old

Any rice consumption was greater in adolescents of rich (98%) and middle (95%) SES than of poor SES (88%). As observed in the younger age groups, consumption of any fish was greater in children of poor SES (68%) than of middle (62%) and rich (54%) SES, with no difference in any vegetable consumption. While any fruit consumption was significantly greater in children of poor and middle SES (19%) than of rich SES (15%) (*p* > 0.05). Consumption of any eggs, any pork, any sausages, and any chicken was greater in adolescents of rich and middle SES, as was consumption of any milk and any sugar-sweetened beverages (*p* > 0.05).

### 3.3. Energy Contribution

The contribution of foods to energy intake by wealth status is presented in Table 4. Rice contributed to 56% of the energy intake in 6–9-year-old children from the poor households, 49% in middle and 43% in the rich households. Corn grits contributed to 4.2% of energy intake in the poor, 2% in the middle households, with a lower contribution in the rich households (0.2%). Fish presented a greater contribution to energy intake in those of poor SES (4%) than of middle (3%) and rich SES (2.2%). The contribution of pork, chicken and beans, nuts, and peas to energy intake was greater in children of rich SES. A similar pattern was seen among 10–12-year-old children, wherein rice contributed to 60% of energy intake in those of poor SES, 53% of middle, and 49% of intake of rich SES. Corn grits contributed a greater proportion of energy in those of poor SES (5.1%), in comparison to bread (4.7%) and noodles (6.4%), which contributed a greater proportion in those of rich and middle SES. Vegetables had a higher contribution in those of poor SES (2.2%) than of rich SES (1.2%). The contribution of pork was comparable between the SES groups, while chicken and sausages contributed to a greater proportion of energy intake in those of middle and rich SES. The top 10 contributors to energy intake were similar in 10–12 years old and 13–18 years old, where rice contributed the greatest across all SES groups. The contribution from noodles was comparable across SES groups in this age group, whereas the contribution from corn grits (4.8%) was greater in the poor households and bread (4.5%) in the rich households. Protein-containing foods had a greater contribution to intake among children from the wealthiest households.

### 3.4. Food Group Intake: Amounts Consumed

#### 3.4.1. 6–9 Years Old

Food group intake of 6–9-year-old children is presented in Table 5. There were significant differences in consumption of a number of foods according to wealth status, including rice, vegetables, meat, milk, and sweetened beverages. Intake of rice, noodles, and pasta (poor, 205 g/day; middle, 184.3 g/day; rich, 186.5 g/day) and “other grain products”, including corn grits (poor, 116.6 g/day; middle, 121.6 g/ day; rich, 85 g/ day) were significantly greater in children of poor and middle SES (*p* < 0.001). Intake of milk, meat, eggs, and cereals was higher in children of rich SES. Similarly, sweetened beverages (poor, 95.9 g/ day; middle, 108.3 g/ day; rich. 161.8 g/ day) and candy (poor, 14.6 g/ day; middle, 15 g/ day; rich, 19.1 g/ day) were consumed in significantly greater quantities in children of rich SES (*p* < 0.05). However, intake of fruits (poor, 90.6 g/ day; middle, 89.9 g/ day; rich, 70.7 g/ day) and vegetables (poor, 62.5 g/ day; middle, 48.3 g/ day; rich, 50.1 g/ day) were significantly greater in children of poor SES (*p* < 0.01).

#### 3.4.2. 10–12 Years Old

Significant differences were observed in intake of fish, sweetened beverages, meat, bread, and milk by wealth status in 10–12-year-old children (Supplementary Table S1). There was no significant difference in intake of rice, noodles, and pasta according to wealth in this age group, yet intake of “other grain products” was significantly greater in children of poor SES. Intake of fish and shellfish (poor, 60 g/day; middle, 57 g/day; rich, 67.8 g/day), meat (poor, 93.4 g/day; middle, 104.5 g/day; rich, 133.8 g/day), and eggs (poor, 38.1 g/day; middle, 41.9 g/day; rich, 42.1 g/day) was significantly greater in children of rich SES (*p* < 0.05). Milk intake was also significantly greater in children of rich SES (poor, 23.5 g/day; middle, 36.8 g/day; rich, 47.5g/day) (*p* < 0.001), but not significantly different between poor and middle households. As per the 6–9-year-old population, intake of fruit (poor, 106g/day; middle, 96.4 g/day; rich 78.3 g/day) was significantly greater in children of poor SES (*p* < 0.05), while vegetable intake (poor, 77.1 g/day; middle, 58.9 g/day; rich, 65.9 g/day) had no significant difference between wealth categories. Consumption of non-alcoholic beverages, including tea and coffee (poor, 6.7 g/day; middle, 15.8 g/day; rich, 54.7 g/day), and sweetened beverages (poor, 108.5 g/day; middle, 125.7 g/day; rich, 196.7 g/day) was significantly greater in children of rich SES (*p* < 0.001), but not significantly different between poor and middle households.

#### 3.4.3. 13–18 Years Old

The differences in food intake by socio-economic status in 13–18 years old are presented in Supplementary Table S2. Similar to the 6–9-year-old population, rice, noodles, and pasta (poor, 319.3 g/day; middle, 330.2 g/day; rich, 301.1 g/day), and other grain products (poor, 180.8 g/day; middle, 196.5 g/day; rich, 112.3 g/day) were significantly greater in adolescents of poor SES than of richer SES (*p* < 0.001), but not between poor and middle households. Similar to the younger populations, intake of fish (poor, 69.5 g/day; middle, 75.2 g/day; rich, 74 g/day) was significantly lower in poor households (*p* < 0.01), and meat (poor, 103.5 g/day; middle, 118.3 g/day; rich, 150.2 g/day) was significantly greater in those of rich SES (*p* < 0.01). While intake of fruit (poor, 120.2 g/day; middle, 105.6 g/day; rich, 87.4 g/day) and vegetables (poor, 90.6 g/day; middle, 74.1; rich, 87.4 g/day) was greater in those of poor SES (*p* < 0.001) compared to middle and rich SES. There were notable differences in beverage intake, wherein adolescents of rich SES had greater intake of milk (poor, 17.1 g/day; middle, 31 g/day; rich, 45.5 g/day), non-alcoholic beverages (poor, 10.2 g/day; middle, 22.9 g/day; rich, 39.5 g/day), and sweetened beverages (poor, 134.6 g/day; middle, 170.7 g/day; rich, 220.1 g/day). There were no differences in candy or ice-cream intake according to wealth status; however, intake of savory snacks (poor, 30.6 g/day; middle, 34.8 g/day; rich, 42.2 g/day) were greater in adolescents of rich SES (*p* < 0.001).

## 4. Discussion

A greater variety of foods were consumed by children of rich SES; nonetheless, rice was the greatest contributor to energy intake in all age groups, independent of wealth status. The prevalence of stunting was greater in Filipino children, of all ages, of the poorest SES, while the prevalence of being overweight was greater in children of middle and rich SES [1]. This double burden of malnutrition is of major public health concern because of its immense impact on the economy at the individual, community, and the population at-large levels, perpetuating the cycles of poverty and ill-health. It has been estimated that the global economy loses US$2 trillion per annum, while undernutrition and micronutrient deficiencies account for an additional US$2.1 trillion. In the Philippines, it was computed that the losses due to undernutrition alone were Php.220 billion per year. Therefore, effective public health strategies, promoting a varied, balanced diet, are required to improve dietary quality and combat this increasing prevalence of malnutrition, especially among those of poor SES.

### 4.1. Lack of Dietary Diversity as the Root of Inadequate Nutrient Intake

The nutrient intake of this young Filipino population have been recently published [4]. In general, Filipino children and adolescents presented inadequate intake of protein, fat, calcium, iron, vitamin C, and B vitamins. Greater inadequacies were observed in children of poorer SES, with the exception of vitamin C [4]. This study highlighted low intake of milk and meat in all children of poor SES across the three age groups; in contrast, intake of vegetables and fruit, which included fruit juice, were greater in children of poor SES. However, the overall consumption of fruit and vegetables in the Philippines is low compared to other countries [21]. Cumulatively, these results may help to explain the observed inadequacies in nutrient intake; however, independent of wealth status, with the exception of rice, fish, and fats and oils, the majority of foods were consumed by less than 60% of each population sub-group, with less than 30% reporting consumption of milk and protein-containing foods. Furthermore, a recent study by our group applied a diet diversity score (DDS) to assess the diet diversity of Filipino school-aged children (6–12 years). In brief, the average DDS of Filipino children was 4/9, with SES having a significant impact on the score, wherein children from poorer households presented the lowest scores. This analysis also assessed the probability of micronutrient adequacy, and found that regardless of the DDS score, Filipino children found it difficult to achieve adequacy in a number of key micronutrients, including calcium and iron. An explanation for this is the small quantities of nutrient-rich foods consumed [22].

Dwelling location and SES both impact fruit and vegetable consumption; intake is typically greater in rural than urban locations in the Philippines—however, this intake remains low [21]. In the current study, we assessed the impact of SES on food group intake of children. Typically, 6–9-year-old children of poor SES consumed 90 g, which is a little over 1 portion of fruit (80 g), compared to 70 g per day in those of middle and rich SES. Adolescents of poor SES consumed 1.5 portions (120 g), compared to 87 g in those of rich SES. Vegetable intake was lower, ranging from 50–70 g in 6–12-year-old children. This may be due to limited access to fruit and vegetables, cost impediments, or a lack of nutrition knowledge [23]. In our recent publication, fiber intake was greater in children from rural dwelling locations; this may be a result of a significantly greater vegetable intake in these children [4]. Intake of fruit and vegetables increased with increasing age by approximately 20 g per day. In contrary, children of poor SES presented low consumption of protein-containing foods, with the exception of fish; this was reflected in their protein, iron, and B vitamin intake, whereby children of poor SES presented the greatest inadequacies [4]. This is likely due to inadequate access to food or cost restrictions [2].

Dietary diversity in the Philippines is similar to that reported in other developing countries, despite the influence of local culture on habitual dietary intake. A limited number of studies have reported on the influence of wealth status on food sources in children from developing countries. Ahmed et al. reported that adolescents from poorer backgrounds in Bangladesh consumed a less diverse diet, and compared to adolescents from a richer background, they presented lower intake of eggs, milk, and fruit [6]. This study was conducted in an urban location, which may explain the difference in fruit consumption to the current study, as the majority of children of poor SES lived in a rural location, which may have improved access to fresh fruit. A more recent study in Brazil demonstrated a higher intake of milk and processed meats, and lower intake of fruit and vegetables, in children of richer SES than of poorer SES, which is comparable to the findings in the current study [3]. While dietary diversity was not quantitatively measured using a score in the current study, a similar pattern emerged, wherein consumption of a less variety of foods was associated with increased nutrient inadequacies [4]. A recent study in Chinese children and adolescents found that children who consumed a greater variety of foods presented lower nutrient inadequacies [24]. This was comparable to the findings in younger Filipino children [5]. Similarly, a stepwise decrease in the prevalence of inadequacies was observed with increasing SES in the current study, with a greater variety of foods consumed by the children and adolescents of rich SES. Hence, public health strategies are required to promote increased consumption of nutrient-rich foods to improve nutrient inadequacies in Filipino children.

### 4.2. Food Insecurity

There is a prevalence of energy inadequacy in the current population, with 19% of 6–9 years old, 29% of 10–12 years old, and 35% of 13–18 years old presenting a mean intake below the EER (estimated energy requirement) [4]. Food insecurity is one of the causative factors, due to low consumption of a variety of foods. A 2013 survey highlighted that 65% of Filipino families are affected by food insecurity, with greater impact observed in rural locations and those of poorer SES, with the staple diet being comprised predominantly of refined rice [2]. This is evident in the current study, wherein the top contributor to energy intake was rice, the local staple in the Philippines, contributing to between 42 and 52% of intake in children and adolescents of rich SES, 49–60% of middle, and 56–62% of poor SES, followed by fats and oils (2–4%). A study in 6–9-year-old children in India reported that cereals (including rice) contributed to 35% of energy intake, and fats and oils to 30%, highlighting the important role of rice as an energy source in the diets of Filipino children [25]. The remainder of energy intake in those of poor SES in the current study typically came from corn grits and fish, and from bread, pork, chicken, and sausages in those of middle and rich SES. However, regardless of age and wealth status, after rice, the remaining food sources of energy contributed to less than 10% of energy intake in the current population, with increased diversity observed in those of middle and rich SES. Cookies contributed to 2.2% of energy intake in 6–9 years old of rich SES, while vegetables contributed to 2.2% of energy intake of older children of poor SES. A review by Ochala et al. (2014) characterized the energy and nutrient intake of school-aged children in developing countries and found that the majority of school-aged children presented inadequate energy and nutrient intake, with low contributions from fruit and vegetables and increasing contributions from sweets and sugary beverages, particularly in urban areas, as observed in the current study [25]. A greater variety of foods was consumed by children and adolescents in the rich households, which is similar to what has been reported in other developing countries [26]. However, the quantities consumed were typically low, which explains why inadequacies in certain nutrients are still prevalent in children of rich SES, despite consuming a more varied diet [4].

### 4.3. Food Fortification of Commonly Consumed Foods

One strategy to improve the nutritive value of commonly consumed foods includes fortification. Rice, the staple food of Filipinos, consumed by almost 98%, is fortified with iron [27], since the prevalence of iron inadequacy ranges from 66 to 92% in 6–12-year-old Filipino children, depending on SES [4]. Therefore, strategies are required to improve the reach of fortified rice. Currently, the Department of Science and Technology—Food and Nutrition Research Institute continuously transfers the technology to more millers and investors across the country. This activity is coupled with social marketing, tapping both millers/investors and the community as the users. Iron is a vital nutrient for growth and development in this age group [28]. Another commonly consumed food is orange juice. Previous work by our group has demonstrated that fortifying orange juice with iron is an effective strategy to reduce the prevalence of iron anemia in school-aged children [29]. Currently, the majority of orange juice is fortified with iron and vitamin C in the Philippines. As fruit consumption (including fruit juice) was higher in 6–9-year-old children of poor SES, this may be a contributory factor as to why there was no difference in vitamin C inadequacies between those of poor and rich SES in the current population [4]. Caution, however, must be observed for the amount of added sugars to these fruit juices in order not to create another nutritional problem. Moreover, milk consumption was significantly greater in children and adolescents of rich SES, due to the high cost of milk [30]. The primary type of milk consumed in the Philippines is powdered, which is typically fortified with iron, zinc, and vitamin C; therefore, it is a potential contributing factor in the lower inadequacies of the aforementioned nutrients and calcium in children and adolescents of rich SES. It is evident that effective public health strategies are required to bridge the gap in nutrient inadequacies between the wealth groups in the Philippines, which could include promotion of the consumption of fortified products meeting at least 30% of the recommended intake.

### 4.4. Strengths and Limitations

This study has a number of strengths and limitations. To date, there is no information available on the impact of wealth status on the food source of nutrients in Filipino children. Therefore, this pertinent study provides novel data that could be used to support government initiatives to improve dietary quality. A further strength of the study was the large, nationally representative sample size, including three age segments and data on SES, which facilitated an in-depth analysis on the socio-economic impact on food intake in Filipino children and adolescents. Furthermore, the food groups applied in the study were representative of the local Filipino diet. An expanded Philippine Food Composition Table (Phil FCT) was updated just for this study in order to report more nutrients. The old Philippine FCT contained only 13 nutrients, while the new one contains 27 nutrients. All food items not found in our FCT were matched with other FCTs from other ASEAN countries, but the majority were derived from the USDA FCT. However, this updated FCT has not yet been published. Furthermore, as the study was cross-sectional, one of the limitations is the self-reported dietary intake data, which may have resulted in under- or over-estimation of food intake.

## 5. Conclusions

This study provides important insights into the impact of wealth status on food consumption and food sources of energy in Filipino children and adolescents. The findings demonstrate that children and adolescents of poor SES have a less varied diet than those of rich SES. Rice is the primary source of energy in this population, regardless of age and wealth status. Those of poor SES presented greater consumption of fruit, vegetables, and fish, while those of middle and rich SES presented greater consumption of milk and protein-containing foods. Furthermore, the comparative food choice motives relatively explain this SES influence, and also an additional factor related to dietary intake. These findings complement a recent publication on the nutrient inadequacies in this population, which reported greater inadequacies of many vital nutrients, including protein, iron, and calcium, in those of poor SES. Cumulatively, these results highlight the need for effective strategies to improve food intake, and subsequently, nutritional inadequacies in this young, venerable population. These findings could be used by government agencies like the Department of Agriculture and Department of Education and non-profit organizations in the Philippines for the development of programs, policies, and advocacy initiatives to address food distribution inequalities and access, especially among the poor in the rural areas.

## Figures and Tables

**Table 1:** Food group classification.

1. MILK	28. VEGETABLES	SWEETS and SNACKS
*2. Infant Formula*	*29. Dark Green Leafy Vegetables*	*55. Sweet Bakery Products*
*3. Toddler/Pre-Schooler Formula*	*30. New Zealand Spinach Leaves*	*56. Cookies*
*4. Milk, Fluid, and Powdered ^a^*	*31. Broccoli*	*57. Biscuits/Crackers*
*5. Cheese*	*32. Cabbage, Green*	*58. Sweet Breads*
*6. Yoghurt*	*33. Other Local Leafy, Petioles, and Salad Vegetables ^d^*	*59. Cakes*
**MEATS/POULTRY/FISH/BEANS**	*34. Deep Yellow Vegetables*	*60. Ice Cream and Popsicles*
*7. Beef*	*35. Carrot*	*61. Candy*
*8. Carabeef*	*36. Sweet Potato, Yellow*	*62. Table Sugar*
*9. Pork*	*37. Cassava, Yellow*	*63. Syrup*
*10. Goat/Lamb*	*38. Squash Fruit*	*64. Preserves, Jams, Jellies*
*11. Chicken*	*39. Squash, Summer Fruit*	*65. Native Snacks*
*12. Duck*	*40. Root and Tuberous Vegetables*	*66. Savory Snacks*
*13. Sausages*	*41. Sweet Potato*	**SWEETENED BEVERAGES**
*14. Luncheon Meats*	*42. Potato*	*67. Fruit-Based Beverages*
*15. Cold Cuts (Ham)*	*43. Other Vegetables ^e^*	*68. Concentrated Fruit Juice Drinks*
*16. Fish and Shellfish ^b^*	**FRUITS AND 100% FRUIT JUICE**	*69.Powdered Fruit Juice Drinks/Fruit-Flavored Drinks*
*17. Eggs and Egg Dishes ^c^*	*44. Fruit, Fresh*	*70. Ready to Drink Fruit Juice Drinks*
*18. Beans, Nuts, and Peas*	*45. Apples*	*71. Soft Drinks (Coca-Cola)*
**GRAINS AND GRAIN PRODUCTS**	*46. Avocado*	*72. Chocolate Beverages/Chocolate-Flavored Beverages*
*19. Cereal*	*47. Banana*	*73. Other Sweetened Beverages ^f^*
*20. Bread*	*48. Mango*	**Mixed Dishes**
*21. Crackers*	*49. Melon*	*74. All Mixed Dishes*
*22. Pancakes, Waffles, and French Toast*	*50. Citrus Fruits*	*75. Meat-Based Mixed Dishes ^g^*
*23. Rice*	*51. Cherries and Berries*	*76. Nut/Pea/Bean-Based Mixed Dishes ^h^*
*24. Pasta*	*52. Papaya*	*77. Grain-Based Mixed Dishes ^i^*
*25. Noodles*	*53. Food Fruit, Canned*	*78. Soups*
*26. Corn Grits*	*54. 100% Fruit Juice (Lemon, Mango, Apple, and Pineapple)*	**OTHER**
*27. Cornmeal*		*79. Fats and Oils*
		*80. Condiments, Sauces, Herbs, Spices, and Other Seasonings*

^a^ Includes fortified milk, cow’s milk, and goat’s milk. ^b^ Includes aquatic shell mollusk (e.g., an oyster or cockle) or crustacean (e.g., a crab or shrimp). ^c^ Includes eggs reported separately and eggs included in disaggregated food mixture. ^d^ Includes horseradish tree leaves, pechay leaves, sweet potato leaves, swamp cabbage, taro leaves, etc. ^e^ Includes chayote fruit, eggplant, bamboo shoot, string yard long bean pod, green, corn on cob, white, corn on cob, yellow, etc. ^f^ Includes coffee, creamer, and sugar (3 in 1), iced tea, salabat, lemon tea drink. ^g^ Includes beef meat ball, roast pig, meat pie, meat roll, menudo canned, pork adovo, canned, pork afritada canned, pork and beans, canned. ^h^ Includes green pea, dried, fried and seasoned, green pea–corn–peanut mixture, fried, and lima bean, fried and seasoned. ^i^ Includes champorado pre-mix, rice gruel, thin, rice gruel w/choc and milk, rice gruel w/chicken, spaghetti, meat balls and canned tomato sauce.

**Table 2:** Population demographics and nutritional status split by socio-economic status ^1^.

	6–9 Years			10–12 Years			13–18 Years		
	Poor	Middle	Rich	Poor	Middle	Rich	Poor	Middle	Rich
Sample size (*n*)	1812	649	1030	1581	519	798	2536	1082	1684
**Gender**									
Male	919 (51.4)	332 (18.6)	538 (30.1)	831 (55.1)	248 (16.4)	430 (28.5)	1396 (50)	557 (20)	838 (30)
Female	893 (52.5)	317 (18.6)	492 (28.9)	750 (54)	271 (19.5)	368 (26.5)	1139 (45.4)	525 (20.9)	846 (33.7)
**Region**									
Urban	485 (32.7)	297 (20)	703 (47.3)	413 (34.9)	232 (19.6)	537 (45.4)	678 (29.6)	471 (20.6)	1138 (49.8)
Rural	1327 (66.1)	352 (17.5)	327 (16.3)	1168 (68.1)	287 (16.7)	261 (15.2)	1857 (61.6)	611 (20.3)	546 (18.1)
**Underweight Status**									
Underweight	715 (40)	189 (29.5)	148 (14.7)	-	-	-	-	-	-
Not Underweight	1072 (60)	452 (70.5)	855 (85.2)	-	-	-	-	-	-
**Stunting**									
Stunted	701 (39.3)	159 (24.9)	129 (13)	638 (41.1))	139 (26.9)	130 (16.5)	968 (39.1)	276 (26.2)	320 (19.5)
Not Stunted	1084 (60.7)	480 (75.1)	873 (87)	914 (58.9)	377 (73.1)	658 (83.5)	1509 (60.9)	779 (73.8)	1324 (80.5)
**Wasting**									
Wasted	190 (10.6)	71 (11.1)	67 (6.7)	221 (14.2)	82 (15.9)	108 (13.7)	287 (11.59)	144 (13.7)	158 (9.6)
Not Wasted	1594 (89.3)	568 (88.9)	935 (93.3)	1331 (85.8)	434 (84.1)	680 (86.3)	2189 (88.4)	910 (86.3)	1486 (90.4)
**Overweight Status**									
Overweight	41 (2.3)	24 (3.8))	89 (8.9))	45 (2.9)	26 (5)	98 (12.4)	71 (2.9)	47 (4.5)	135 (8.2)
Not Overweight	1743 (97.7)	615 (96.2)	913 (91.1)	1507 (97.1)	490 (95)	690 (87.6)	2405 (97.1)	1007 (95.5)	1509 (91.8)

^1^ Count (column %) presented in the table.

**Table 3:** Mostly consumed food by age group and socio-economic status ^1^.

	6–9 Years Old			10–12 Years Old			13–18 Years Old		
Food Group	Poor (*n* = 1812)	Middle (*n* = 649)	Rich (*n* = 1030)	Poor (*n* = 1581)	Middle (*n* = 519)	Rich (*n* = 798)	Poor (*n* = 2536)	Middle (*n* = 1082)	Rich (*n* = 1684)
**Any Rice**	89	95	50 ^a,b,c^	87	94	99 ^a,b,c^	88	95	98 ^a,b,c^
**Fats and Oils ^2^**	59	67	38 ^a,b,c^	59	69	78 ^a,b,c^	58	66	72 ^a,b,c^
**Fish**	66	56	26 ^a,b,c^	69	60	57 ^a,b,c^	68	62	54 ^a,b,c^
**Vegetables**	53	49	23 ^a,b,c^	59	55	48 ^a,b,c^	60	59	57 ^NS^
**Seasonings ^3^**	32	28	18 ^b,c^	31	32	38 ^b,c^	32	34	41 ^b,c^
**Eggs**	22	31	17 ^a,b,c^	20	30	33 ^a,b^	19	30	32 ^a,b^
**Table Sugar**	29	27	12 ^b,c^	27	26	22 ^b^	28	26	21 ^b,c^
**Bread**	22	28	18 ^a,c^	20	33	35 ^a,b^	20	26	32 ^a,b,c^
**Pork**	14	24	19 ^a,b,c^	15	26	40 ^a,b,c^	18	26	44 ^a,b,c^
**Noodles**	22	25	11 ^b,c^	20	26	21 ^a,c^	22	27	22 ^a,c^
**Fruits**	19	15	7 ^b,c^	19	13	16 ^a^	19	19	15 ^b,c^
**Milk**	9	18	18 ^a,b^	6	14	28 ^a,b,c^	5	11	19 ^a,b,c^
**Chicken**	11	20	16 ^a,b,c^	11	21	31 ^a,b,c^	13	22	36 ^a,b,c^
**Sausages**	7	19	15 ^a,b,c^	8	20	28 ^a,b,c^	7	15	22 ^a,b,c^
**Soft Drinks**	4	7	8 ^a,b^	6	12	20 ^a,b,c^	10	18	27 ^a,b,c^

^1^ Values are percentage of children consuming the food category by wealth status during a single 24-h recall. ^2^ Includes oils, fats (from plant and animal source), fats and oil products. ^3^ Includes condiments, pasta/pizza sauces, herbs and spices, and other seasoning flavoring extracts. ^a^ Poor vs. middle, ^b^ poor vs. rich, ^c^ middle vs. rich, significantly different if *p*-value <0.05 (independent sample *t*-test for two proportions with Bonferroni multiple comparison adjustment).

**Table 4:** Top 10 food sources of energy by age group and socio-economic status ^1^.

	6–9 Years Old			10–12 Years Old			13–18 Years Old		
	Poor (*n* = 1812)	Middle (*n* = 649)	Rich (*n* = 1030)	Poor (*n* = 1581)	Middle (*n* = 519)	Rich (*n* = 798)	Poor (*n* = 2536)	Middle (*n* = 1082)	Rich (*n* = 1684)
Rice	56	49	43	60	53	49	62	60	52
Corn Grits, White	4	2	0	5	2	0	5	2	0
Bread	4	6	5	3	6	5	3	4	5
Fish and Shellfish	4	3	2	4	3	3	3	3	3
Noodles	3	4	4	3	4	3	4	4	3
Pork	3	3	6	3	4	6	3	4	8
Fats and Oils	3	3	3	2	3	4	2	3	3
Cookies	1	2	3	-	-	-	-	-	-
Sausages	1	3	4	1	3	4	1	2	3
Chicken	1	2	3	1	2	3	1	2	3
Vegetable	-	-	-	2	1	1	2	1	1

**^1^** Percent contribution of each food group to the total energy intake during a single 24-h recall.

**Table 5:** Food group intake (grams) in 6–9-year-old children (consumers only), split by socio-economic status (mean and standard error).

	Poor	Middle	Rich	
	(*n =* 1812)	(*n =* 649)	(*n =* 1030)	*p*-value ^†^
Rice, Noodles, and Pasta ^1^	205 (2.9)	184.3 (4.1)	186.5 (3.3)	0.000 ^a,b^
Bread, Rolls, and Biscuits	55.5 (2.1)	63.6 (3.1)	62.1 (2.3)	0.004 ^a,b^
Cereals	24.2 (1.9)	33.2 (4.9)	33.7 (2.5)	0.009 ^b^
Savory Snacks ^2^	22.5 (1.3)	20.7 (1.4)	30.9 (1.8)	0.000 ^b,c^
Grain-Based Mixed Dishes ^3^	240.5 (15.8)	213.2 (13.2)	218.4 (11.3)	0.471
Other Grain Products ^4^	116.6 (4.3)	121.6 (15.4)	85 (19.7)	0.002 ^b,c^
Vegetables	62.5 (3)	48.3 (3)	50.1 (2.6)	0.01 ^a^
Fruits	90.6 (4.5)	89.8 (9.4)	70.7 (6.3)	0.000 ^b,c^
Fish and Shellfish ^5^	51.3 (1.4)	51.1 (2.8)	52.5 (2.1)	0.071
Meat	87.7 (3.2)	86.4 (3.6)	106.2 (2.9)	0.000 ^b,c^
Egg and Egg Dishes	36.7 (1.3)	40.5 (1.6)	40.4 (1.4)	0.025^a^
Beans, Nuts, and Peas	35.2 (3.6)	37.5 (5.1)	35.7 (5.6)	0.582
Nut/Pea/Beans-Based Mixed Dishes ^6^	13.8 (1.7)	23.3 (13.3)	20 (10)	0.572
Milk ^7^	24.2 (2)	28.7 (3.6)	45.7 (3)	0.000 ^b,c^
Non-Alcoholic Beverages ^8^	12.9 (4.5)	13.2 (9.6)	23.8 (7.8)	0.092
Sugar-Sweetened Beverages ^9^	95.9 (5.8)	108.3 (7.7)	161.8 (6.9)	0.000 ^a,b,c^
Sweet Bakery Product ^10^	48.9 (1.7)	48.3 (2.4)	51.9 (1.9)	0.096
Candy	14.6 (1.4)	15 (1.4)	19.1 (1.5)	0.036 ^b^
Native Dessert Snacks ^11^	86.1 (4.8)	107.8 (8.6)	108.4 (11.3)	0.023b
Ice Cream and Popsicles	116.9 (7.3)	105 (7.1)	128.4 (11.6)	0.477
Pancakes, Waffle, and French Toast	51.6 (4.5)	49.1 (7.7)	70 (8.8)	0.122
Other Dessert Snacks ^12^	25.9 (7.1)	40.1 (8.5)	29.4 (3.7)	0.121
Fat ^13^	11 (0.8)	10.1 (1.5)	10.6 (0.6)	0.186
Fats and Oils Products ^14^	8.3 (1.5)	8.3 (2.9)	12.2 (1.6)	0.289
Table Sugar, Syrup, Preserve Jams and Jellies	10.2 (0.4)	9.7 (0.7)	11.1 (0.7)	0.481
Seasonings ^15^	8.6 (0.4)	10.1 (0.9)	14.1 (1.2)	0.000 ^b,c^
Flour, Cornstarch, Baking Powder, and Yeast	11.8 (4)	25.8 (17.8)	36.8 (7.6)	0.248
Miscellaneous Food Items ^16^	8 (1.8)	5.7 (1.1)	12.8 (2.2)	0.003 ^b,c^

^1^ Includes rice (also rice products), pasta, and noodles. ^2^ Includes potato-based, corn tortillas, prawn/fish crackers, curls, and puffs. ^3^ Includes champorado pre-mix, rice gruel, spaghetti, meatballs and tomato sauce, canned. ^4^ Includes corn grits, cornmeal, Job’s tears grain, and millet glutinous. ^5^ Includes fresh, dried, cooked, smoked, canned fish and fish products. ^6^ Includes green pea, dried, fried and seasoned, fries green pea–corn–peanut mixture, and Lima bean, fried and seasoned. ^7^ Includes fluid and powdered milk, dairy products, and other milk. ^8^ Includes tea, coffee, cocoa powder, and coconut water. ^9^ Includes fruit-based, chocolate/chocolate-flavored, soy, prepared sweet, and other sweetened beverages and soft drinks. ^10^ Includes cookies, biscuit/crackers, sweet breads, cakes, pies, and other pastries. ^11^ Includes cassava, banana, mango snacks, and other snacks. ^12^ Includes chocolate creams, chocolate, tablea, coconut meat, peanut brittle, polvoron, popcorn, etc. ^13^ Includes coconut cream, margarine, fat from pork and beef. ^14^ Includes sandwich spread, all-purpose dressing, mayonnaise, coconut cream curd, etc. ^15^ Includes condiments, pasta/pizza sauces, herbs and spices, and other seasoning flavoring extracts. ^16^ Includes coffee creamer, food coloring, gelatin powder, spring roll wrapper, strained, juice from duodenum, locust, bird’s nest, azolla pinnata, and vegemeat, textured wheat and soy protein. ^†^ significantly different if *p*-value <0.05, ^a^ poor vs. middle, ^b^ poor vs. rich, ^c^ middle vs. rich (One-way ANOVA with Bonferroni multiple comparison test).

## Supplementary Materials

The following are available online at https://www.mdpi.com/2072-6643/11/12/2910/s1, Table S1. Food group intakes (grams) in 10–12 years old children, split by socio-economic status (mean and standard error), Table S2. Food group intakes (grams) in 10–12 years old children, split by socio-economic status (mean and standard error).
