# Supplementary material for: The Impact of Wealth Status on Food Intake Patterns in Filipino School-Aged Children and Adolescents
Source: Nutrients. 2019 Dec 2;11(12):2910. doi: 10.3390/nu11122910 (PMC6950240; doi:10.3390/nu11122910)
Supplement: Supplementary file 1 [file nutrients-11-02910-s001.pdf]

Supplementary Table 1. Food group intakes (grams) in 10-12 year old children, split by socio-economic status (mean and SE)

Food Groups	Poor (*n*=1581)	Middle (*n*=519)	Rich (*n*=798)	*p-value*†
Rice, Noodles, & Pasta[1]	265.5 (3.7)	251.8 (6)	255.2 (4.8)	0.239
Bread, Rolls, & Biscuits	59.2 (2.3)	70.9 (3.6)	65.1 (2.7)	0.002[a]
Cereals	29.3 (2.8)	30.3 (1.3)	42.9 (4.8)	0.04[b]
Savory Snacks[2]	24.7 (1.4)	33.5 (4.3)	35.8 (3)	0.000[b]
Grain Based Mixed Dishes[3]	250.7 (16.1)	257.7 (17.4)	223.5 (11.4)	0.109
Other Grain Products[4]	157.5 (6)	152 (15)	79.4 (14.3)	0.000[b,c]
Vegetables	77.1 (2.9)	58.9 (3.2)	65.9 (3.5)	0.049
Fruits	106 (6.9)	96.4 (11.8)	78.3 (6.7)	0.000[b]
Fish and Shellfish[5]	60 (1.6)	57.3 (2.6)	67.8 (2.6)	0.001[b,c]
Meat	93.4 (4)	104.5 (4.7)	133.8 (3.9)	0.000[b,c]
Egg & Egg Dishes	38.1 (1.4)	41.9 (2.1)	42.1 (1.5)	0.036[b]
Beans, Nuts, & Peas	50.5 (6)	51.1 (11.6)	59 (10.5)	0.452
Nut/Pea/Beans-Based Mixed Dishes[6]	16.4 (3.5)	33.3 (18.5)	26 (24)	0.5
Milk[7]	23.5 (2.6)	36.8 (9)	47.5 (4.6)	0.000[b,c]
Non Alcoholic Beverages[8]	6.7 (2)	15.8 (7)	54.7 (17.1)	0.000[b,c]
Sugar-sweetened Beverages[9]	108.5 (6.5)	125.7 (10.1)	196.7 (8.9)	0.000[b,c]
Sweet Bakery Product[10]	50.8 (2.6)	46 (2.6)	55.6 (2.8)	0.05
Candy	15.3 (1.9)	16.7 (2.1)	19.1 (2.1)	0.215
Native Dessert Snacks[11]	106.3 (8.1)	98.9 (7.1)	91.1 (4.6)	0.841
Ice Cream and Popsicles	105.4 (5.7)	126.3 (12.6)	166.1 (20.7)	0.055
Pancakes, Waffle, & French Toast	52.2 (6.7)	73.8 (17.7)	69.2 (7.7)	0.069
Other Dessert Snacks[12]	24.7 (5.7)	31.9 (5)	30.6 (8.6)	0.574
Fat[13]	13.7 (1.6)	9.8 (1.5)	18.9 (4.2)	0.096
Fats & oils Products[14]	7.9 (1.6)	11.6 (1.4)	8.1 (1)	0.151
Table sugar, syrup, preserve jams & jellies	10 (0.3)	11.8 (0.9)	14.4 (1.4)	0.004[b]
Seasonings[15]	9.7 (0.4)	12.5 (1)	15.6 (1.1)	0.000[a,b]
Flour, Cornstarch, Baking Powder, & Yeast	14.8 (6.5)	26.1 (5.3)	30.1 (6.2)	0.479
Miscellaneous Food Items[16]	6.6 (0.5)	11.3 (1.7)	11.2 (1.4)	0.002[a,b]

[1]includes rice (also rice products), pasta, and noodles
[2]includes potato based, corn tortillas, prawn/fish crackers, curls and puffs
[3]includes Champorado Pre-Mix, Rice gruel, Spaghetti, meat balls & tomato sauce, canned
[4]includes corn grits, cornmeal, Job's tears grain, and Millet glutinous
[5]includes fresh, dried, cooked, smoked, canned fish and fish products
[6]includes Green pea, dried, fried & seasoned, Fries green pea-corn-peanut mixture, and Lima bean, fried & seasoned
[7]includes fluid and powdered milk, dairy products, and other milk
[8]includes tea, coffee, cocoa powder, and coconut water
[9]includes fruit-based, chocolate/chocolate flavored, soy , prepared sweet, and other sweetened beverages and softdrinks
[10]includes cookies, biscuit/crackers, sweet breads, cakes, pies, and other pastries
[11]includes cassava, banana, mango snacks and other snacks
[12]includes Chocolate Creams, Chocolate, tablea, Coconut meat, Peanut brittle, Polvoron, popcorn, etc.
[13]includes coconut cream, margarine, fat from pork and beef
[14]includes sandwich spread, all-purpose dressing, mayonnaise, coconut cream curd etc.
[15]includes condiments, pasta/pizza sauces, herbs and Spices, and other seasonings flavoring extract
[16]includes coffee creamer, food coloring, gelatin powder, spring roll wrapper, strained, juice from duodenum, locust, bird's nest, azolla pinnata, and vegemeat, textured wheat & soy protein
†significantly different if p-value < 0.05, [a]poor vs middle, [b]poor vs rich, [c]middle vs rich (Oneway ANOVA with Bonferroni multiple comparison test)

Supplementayry Table 2. Food group intakes in 13-18 year old children, split by socio-economic status (mean and SE)

	Poor	Middle	Rich	
**Food Groups**	**(*n*=2536)**	**(*n*=1082)**	**(*n*=1684)**	***p-value*†**
Rice, Noodles, & Pasta[1]	319.3 (3.6)	330.2 (5.2)	301.1 (4)	0.000[b,c]
Bread, Rolls, & Biscuits	68.9 (2.2)	66.4 (2.4)	75.9 (2.1)	0.001[b,c]
Cereals	43 (12.5)	28.6 (2.8)	37.9 (4.7)	0.607
Savory Snacks[2]	30.6 (1.4)	34.8 (1.9)	42.2 (2.7)	0.000[a,b]
Grain Based Mixed Dishes[3]	273.7 (35.6)	324.3 (49)	324.6 (28.9)	0.565
Other Grain Products[4]	180.8 (5.1)	196.5 (15.4)	112.3 (15.3)	0.000[b,c]
Vegetables	90.6 (2.5)	74.1 (3.2)	69 (2.6)	0.000[a,b]
Fruits	120.2 (5.6)	105.6 (7.5)	87.4 (6.6)	0.000[a,b]
Fish and Shellfish[5]	69.5 (1.5)	75.2 (2.4)	74 (2.2)	0.004[a,b]
Meat	103.5 (3.1)	118.3 (4)	150.2 (3.2)	0.000[a,b,c]
Egg & Egg Dishes	42.7 (1.3)	40.7 (1.6)	42.4 (1.1)	0.277
Beans, Nuts, & Peas	40.9 (2.4)	43.9 (3.7)	57.2 (6.2)	0.186
Nut/Pea/Beans-Based Mixed Dishes[6]	21.3 (7.7)	12.2 (8.8)	23.3 (6.6)	0.46
Milk[7]	17.1 (1.9)	31 (4.2)	45.5 (4)	0.000[a,b,c]
Non Alcoholic Beverages[8]	10.2 (2.1)	22.9 (5.6)	39.5 (6.9)	0.000[b,c]
Sugar-sweetened Beverages[9]	134.6 (5.7)	170.7 (7.9)	220.1 (6.4)	0.000[a,b,c]
Sweet Bakery Product[10]	60.3 (3)	56.3 (3.9)	58.8 (2.8)	0.968
Candy	16.3 (1.7)	16 (2.5)	17 (1.8)	0.955
Native Dessert Snacks[11]	116.2 (8.3)	116.2 (6.2)	117.2 (8.5)	0.588
Ice Cream and Popsicles	117 (9.3)	116.7 (12.1)	97.6 (7.7)	0.092
Pancakes, Waffle, & French Toast	79.1 (9)	84.3 (15.7)	68.8 (5.3)	0.873
Other Dessert Snacks[12]	33.5 (8.7)	26.7 (4.6)	28.7 (4)	0.87
Fat[13]	13.4 (0.7)	17.6 (3.1)	13.8 (1.2)	0.951
Fats & oils Products[14]	11.1 (3.2)	9.1 (1.7)	9.3 (0.8)	0.739
Table sugar, syrup, preserve jams & jellies	11.7 (0.4)	12.5 (0.7)	13.4 (0.6)	0.473
Seasonings[15]	11.2 (0.5)	13.5 (0.8)	17.2 (0.8)	0.000[a,b,c]
Flour, Cornstarch, Baking Powder, & Yeast	14.5 (3.1)	25.8 (6.9)	38.2 (7.1)	0.045[b]
Miscellaneous Food Items[16]	8.4 (0.8)	10.4 (1.1)	11.2 (0.9)	0.002[b]

[1]includes rice (also rice products), pasta, and noodles
[2]includes potato based, corn tortillas, prawn/fish crackers, curls and puffs
[3]includes Champorado Pre-Mix, Rice gruel, Spaghetti, meat balls & tomato sauce, canned,
[4]includes corn grits, cornmeal, Job's tears grain, and Millet glutinous
[5]includes fresh, dried, cooked, smoked, canned fish and fish products
[6]includes Green pea, dried, fried & seasoned, Fries green pea-corn-peanut mixture, and Lima bean, fried & seasoned
[7]includes fluid and powdered milk, dairy products, and other milk
[8]includes tea, coffee, cocoa powder, and coconut water
[9]includes fruit-based, chocolate/chocolate flavored, soy , prepared sweet, and other sweetened beverages and softdrinks
[10]includes cookies, biscuit/crackers, sweet breads, cakes, pies, and other pastries
[11]includes cassava, banana, mango snacks and other snacks
[12]includes Chocolate Creams, Chocolate, tablea, Coconut meat, Peanut brittle, Polvoron, popsorn, etc.
[13]includes coconut cream, margarine, fat from pork and beef
[14]includes sandwich spread, all-purpose dressing, mayonnaise, coconut cream curd etc.
[15]includes condiments, pasta/pizza sauces, herbs and Spices, and other seasonings flavoring extract
[16]includes coffee creamer, food coloring, gelatin powder, spring roll wrapper, strained, juice from duodenum, locust, bird's nest, azolla pinnata, and

vegemeat, textured wheat & soy protein

[†]significantly different if p-value < 0.05, [a]poor vs middle, [b]poor vs rich, [c]middle vs rich (Oneway ANOVA with Bonferroni multiple comparison test)
